# Community-based dengue control intervention in Ouagadougou: intervention theory and implementation fidelity

**DOI:** 10.1186/s41256-018-0078-7

**Published:** 2018-08-03

**Authors:** Diane Saré, Dennis Pérez, Paul-André Somé, Yamba Kafando, Ahmed Barro, Valéry Ridde

**Affiliations:** 1 University of Montreal Public Health Research Institute (IRSPUM), University of Montreal School of Public Health (ESPUM), 7101 Avenue du Parc, Room 3060, Montreal, H3N 1X9 Quebec Canada; 2 Association AGIR, Ouagadougou, Burkina Faso; 3 IRD (French Institute for Research on Sustainable Development), CEPED (IRD–Université Paris Descartes), Universités Paris Sorbonne Cités, ERL INSERM SAGESUD, Paris, France; 4 Institute of Tropical Medicine Pedro Kourí, Autopista Novia del Mediodia Km 6 1/2, PO Box 601, La Lisa, Marianao 13, Havana City, Cuba

**Keywords:** Community intervention, Dengue, Fidelity and adaptation, Urban setting, Ouagadougou

## Abstract

**Background:**

While malaria control is the primary health focus in Burkina Faso, the recent dengue epidemic calls for new interventions. This paper examines the implementation fidelity of an innovative intervention to control dengue in the capital Ouagadougou.

**Methods:**

First we describe the content of the intervention and its theory. We then assess the fidelity of the implementation. This step is essential as preparation for subsequent evaluation of the intervention’s effectiveness. Observations (*n* = 62), analysis of documents related to the intervention (*n* = 8), and semi-structured interviews with stakeholders (*n* = 18) were conducted. The collected data were organized and analyzed using QDA Miner. The theory of the intervention, grounded in reported good practices of community-based interventions, was developed and discussed with key stakeholders.

**Results:**

The theory of the intervention included four components: mobilization and organization, operational planning, community action, and monitoring/evaluation. The interactions among these components were intended to improve people’s knowledge about dengue and enhance the community’s capacity for vector control, which in turn would reduce the burden of the disease. The majority of the planned activities were conducted according to the intervention’s original theory. Adaptations pertained to implementation and monitoring of activities.

**Conclusions:**

Despite certain difficulties, some of which were foreseeable and others not, this experience showed the feasibility of developing community-based interventions for vector-borne diseases in Africa.

## Background

Dengue fever is a rapidly expanding febrile disease throughout the world. Not only has its prevalence increased considerably in endemic regions, but it is spreading into areas where the viruses responsible for the disease did not previously exist [1, 2]. It is a major public health problem for most tropical and subtropical cities [3]. In Burkina Faso, prior to the dengue fever epidemic in 2013, this disease was almost unknown to both health workers and the greater public, most cases having been considered malaria and treated as such [4]. Studies conducted in the cities of Kaya and Ouagadougou have confirmed the presence of dengue in Burkina Faso [5]. A survey conducted between 2013 and 2014 in six health and social promotion centres (CSPSs) in the city of Ouagadougou showed that 8.7% of rapid diagnostic tests (RDT) performed on febrile patients were positive for dengue fever. The results of this same investigation also revealed the presence of three serotypes of dengue fever virus (DENV2, DENV3, DENV4) [6], which increases the risk of contracting the disease and manifesting the severe form, since cross immunity is only partial and temporary [7].

The absence of specific treatment and the high cost of this disease make prevention the only means of control for the time being in the context of Burkina Faso [6]. However, the prevention of vector-borne diseases remains very poorly developed, and prevention efforts are mainly limited to the distribution of long-lasting insecticide-treated bed nets (LLINs) and some indoor residual spraying (IRS) initiatives in certain endemic areas [8].

Community interventions have proven to be effective in combating dengue fever around the world [9, 10]. However, in Burkina Faso, no community intervention against dengue fever has yet been implemented to our knowledge. Thus, this article aims to describe the development of the first such intervention in Burkina Faso and to analyze its content and the fidelity of its implementation.

### Development of the content of the community intervention against dengue fever

#### Intervention site

The intervention was implemented in Ouagadougou, the country’s capital. It is part of a seroprevalence study conducted at five sites in the capital [11]. The intervention site was chosen on the basis of similarities in socio-environmental characteristics related to febrile diseases and the presence of vectors. Thus, out of five potential intervention zones, two were selected. The intervention was randomly assigned to sector 16 (Tampouy). Sector 2 (Juvénat) was selected as the comparison area. All activities took place within a 1-km radius of the CSPS in Tampouy over a six-month period. The intervention area was divided into four zones: Tampouy-Bilbalgo, Yitouni, Cité An IV B, and Cité Azimo. This division, based on knowledge of the local community, was intended to facilitate the execution of activities and thus allow a better coverage of the intervention area.

#### Selection of activities

The activities to be carried out as part of this intervention were selected based on a review of the scientific literature and taking into account community preferences through a mixed-method study (quantitative–qualitative), the results of which will be the subject of another article. For this study, a questionnaire survey was conducted among households, as well as seroprevalence surveys [11]. The questionnaire covered the following elements: neighbourhood and community life, community preferences in choosing activities, needs to be met, medical and clinical history, daily mobility, budget and time, clinical evaluation for a recent feverish episode, and results of rapid diagnostic tests for malaria. A total of 3066 people responded to the questionnaire. Then, to deepen these quantitative results, 15 focus groups were carried out encompassing a total of 216 individuals (women, men, and youths). They provided opinions and perceptions about community work, their preferences for strategies and actions to be undertaken, the relevance of these strategies and actions, and factors that might be barriers to implementing a community-based intervention in the urban context. The synthesis of the data from these two surveys and a review of the scientific literature enabled the community stakeholders and the AGIR team (*Action, Gouvernance, Intégration, Renforcement* – a Burkinabè non-governmental organization) to discuss and choose a list of activities that were acceptable, appropriate, and potentially effective. This list was presented at a workshop with a small group of 22 people including community leaders, community-based health workers (CHWs), association leaders, and sector-based health workers for the final selection of activities to be implemented. The members of this group were chosen on the basis of their involvement with and commitment to the cause of their community and their capacity for mobilization.

#### Conduct of activities

To carry out activities in the field, the various actors in the intervention area (community leaders, association members, and CHWs) appointed people to act as facilitators. The selected individuals were trained and equipped to conduct the field activities (see Table 1).

**Table 1:** The implementation actors and their role

Actors	Composition	Role
Oversight committee	• Traditional chiefs • Association managers • Religious leaders • Representative of the CSPS • Representative of the association AGIR	Monitor the progress of activities carried out in the field and provide support for any difficulties that might arise
Facilitators	• Designated members of formal and informal associations • Community health workers	Carry out the activities planned as part of the community intervention
AGIR workers	• Intervention coordinator • Coordinator’s assistant	Provide technical support
University of Montreal master’s student	• Trainee/intern	Provide technical support

Two types of activities were carried out, aimed at: 1) increasing the community’s knowledge and changing behaviours: educational talks, theatrical performances, drawing contests, and posters; and 2) destroying breeding sites: door-to-door visits and community-based activities.

Various tools were developed and used: a guide with educational discussion techniques, a box of pictures, dengue awareness posters, and activity report cards. The educational talks involved facilitating groups of people with different profiles (men, women, youths). The box of pictures illustrating the mode of dengue transmission, appearance of symptoms, treatment, larval breeding sites, and prevention methods was used by the facilitators to raise awareness. Door-to-door canvassing involved going into people’s home to meet the community. The facilitators began by sensitizing the members of the household, and then together they identified and destroyed the breeding sites identified in the concession. The theatrical activity began with a 20-min acting performance followed by audience interaction and a question-and-answer session. Community activities consisted of gathering the community around an activity of common interest, such as cleanliness days in primary schools. Posters with information on dengue fever were placed in strategic points defined beforehand by stakeholders. A drawing contest was organized among schoolchildren. It aimed to increase students’ communication skills, as well as their knowledge of dengue fever and vector control activities, but also to encourage them to pass on the information to their parents.

## Methods

### Evaluation objectives

Most studies of community action against dengue have focused on analyzing their effects [12–14]. Analyses of implementation processes of community-based interventions against dengue [15–17] are scarce. Moreover, there are almost no studies that describe the development and implementation fidelity of community-based interventions against dengue [18], and even less so in Burkina Faso [19]. However, the analysis of implementation fidelity is important, in that it not only ensures that the observed results are linked to the intervention [20, 21], but also, from a formative standpoint, generates ideas for improving the implementation of the intervention. Therefore, the evaluation objective is to analyze the fidelity of the implementation of the community intervention to control dengue fever in Ouagadougou.

### Analysis of implementation fidelity

This analysis is part of an evaluation approach based on intervention theory [22].

To understand the logic of the intervention and its various components, which are essential for fidelity analysis, we first developed the theory of the intervention. The theoretical model was constructed in a participatory manner with the involvement of all stakeholders. Data from several sources (intervention protocol, activity reports) were synthesized to build the preliminary theoretical model. This model was presented to and discussed with the various stakeholders (health professionals, facilitators, researchers) to arrive at a final consensus-based theoretical model.

For this study, we adopted Carroll’s [23] definition of fidelity: “the degree to which an intervention is implemented as intended”. To assess fidelity, we compared what actually happened in the implementation with our previously defined specific descriptors for community interventions against dengue fever (Table 2), using questions formulated based on the description of the intervention. We conducted a qualitative analysis using a single case study design. We used the conceptual framework of Carroll et al. [23] modified by Pérez et al. [18]. This conceptual framework retains the idea of conducting outcome evaluation and component analysis to identify the essential elements of the intervention. Rather than considering compliance as the only measure of faithful implementation, the nature of the adaptations and their influence on the intervention’s effectiveness should also be considered. This framework proposes evaluating the specific descriptors of fidelity and any adaptations. To do this, we need to: 1) have an idea of the intervention’s outcomes; 2) explain how the change theory works; 3) draw on this change theory to formulate specific descriptors of fidelity; and 4) formulate questions based on the description of the intervention.

**Table 2:** Specific descriptors of the intervention

Specific fidelity descriptors for the community intervention against dengue fever	
What: strengthening community knowledge and capacity building on dengue fever and mosquito control with an emphasis on community participation	
How: through awareness-raising for behaviour change based on Bandura’s social cognitive theory: in the training provided to community workers, they will acquire knowledge and attitudes favourable to antivectorial control, and as they conduct activities in their neighbourhood, other members of the community will be encouraged to observe and imitate them	
How often: 1 talk per week per zone (total of 8 talks/zone) 1 door-to-door outing per week per zone (total of 8 door-to-door outings/zone) 1 theatrical performance/month per zone (total of 2 theatrical performances/zone) 1 community activity/zone 22 posters in 17 sites over 4 months	
To whom: the community living within a 1-km radius of the CSPS	
By whom: representatives of the community: community-based health workers, associations, religious and traditional leaders, representatives of AGIR	
Specifications related to context: involvement of the mayor’s office, CSPS, climate, other ongoing interventions	

To ensure all adaptations were detected, especially those that had not been anticipated, we identified specific descriptors of the intervention, based on the intervention theory and inspired by the Tidier checklist [24] (Table 2).

We used multiple sources of evidence (i.e., documents, interviews, observations) to be able to triangulate information and increase the internal validity of the study. Thus, we conducted participant observations during the various activities over a period of 3 months (talks, *n* = 20; door-to-door outings, *n* = 30; theatrical performances, *n* = 9; meetings of the oversight committee, *n* = 3) and systematically took notes. The persons taking part in participant observations were AGIR workers (intervention coordinator, coordinator’s assistant) and the student. Participant observation consisted of looking at the following points: the actual conduct of the activity and the frequency, who conducted the activity, how the activity was carried out, the place where the activity was conducted, the persons who participed to the activity and where they came from, and the contextual elements. Since this work was not an exploratory analysis, and because the research questions were known, we chose to conduct semi-structured interviews, using a guide based on the specific descriptors for the intervention. To increase the representativeness of our results, we used maximum variation sampling [25] to select persons to interview by including all the categories of actors who were involved in implementing the intervention. A total of 18 people were interviewed, including three agents of the health working group (AGIR), 14 facilitators, and one member of the oversight committee. Interviews were conducted in the interviewee’s language of choice; nine were conducted in Mooré (the national language) and nine in French. They were recorded with the consent of the participants and then transcribed. Lastly, we analyzed all the available documents concerning the intervention (*n* = 8), including the intervention protocol, activity reports, minutes of oversight meetings, and training materials, etc.

We constructed a fidelity grid based on the content of the intervention theory. This tool was used to document the presence or absence of programmed activities. All collected data were transferred to QDA Miner qualitative analysis software. We drew on the interview guide and the fidelity analysis grid to develop an analytical framework that guided the organization of the data in QDA Miner. The qualitative analysis was thus part of a framework analysis approach [26]. The information extracted from these various sources was used to fill in the fidelity grid and identify activities according to whether they were carried out as planned, modified or adapted, not carried out, or added. Researcher triangulation was done by discussing the extracted information with a group of four professionals (AGIR workers) involved in different stages of the strategy’s implementation to provide a more complete and nuanced understanding of possible interpretations [27]. If the four workers agreed that a component or subcomponent had been implemented as specified in a given zone, it was classified as implemented as planned. If all agreed that a component or subcomponent had not been implemented, it was classified as not implemented. When any member of the group felt that a component or subcomponent had been modified, it was considered modified (Table 3).

**Table 3:** Fidelity grid for the community intervention against dengue in Ouagadougou

	Activities	Implemented as planned	With adaptations	Not implemented	Added
Mobilization and organization	Identification	X	0	0	0
	Mobilization	0	X	0	0
Operational planning	Validation of activities	X	0	0	0
	Development of tools	X	0	0	0
	Training	X	0	0	0
Community action	Talks	0	X	0	0
	Door-to-door	0	X	0	0
	Theatre performances	0	X	0	0
	Community activities	0	X	0	0
	Posters	X	0	0	0
	Applying insecticide paint	0	0	X	0
	SMS and information videos	0	0	X	0
	Drawing contests	0	0	0	X
Monitoring/ evaluation	Monitoring of activities	0	X	0	0
	Participatory evaluation	0	X	0	0
	Results presentation workshop	0	0	X	0

0 = no activities implemented, no activities not implemented, adapted or added

**X** = activities implemented, not implemented, adapted or added in the four intervention areas

## Results

### Intervention theory

**Fig. 1:**
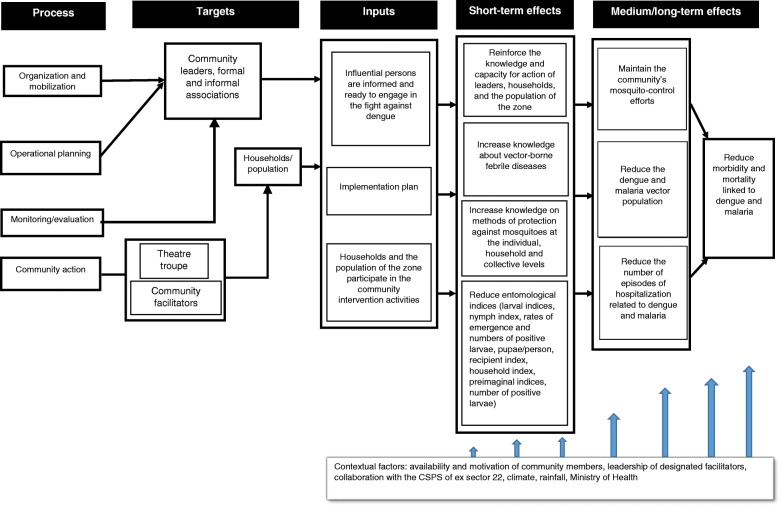
Theoretical model for the community intervention against dengue in Ouagadougou

The theory of the intervention revolved around four components (Fig. 1).

1. Through mobilization and organization, contacts were established with community leaders and with formal and informal associations working in the area, who were given information on the dengue situation in their neighbourhoods. This step helped raise awareness among influential people about the status of this disease and encouraged them to join in combating it.
2. Operational planning consisted of programming the intervention with the participation of influential persons and associations willing to be involved.
3. Community action consisted in community members (facilitators and theatre troupe) carrying out activities for the community. These activities promoted the effective participation of households and the population.
4. The monitoring and evaluation carried out by the community led to greater involvement of the community.

The interaction of these different components had the proximal effects of increasing knowledge on dengue fever and febrile vector-borne diseases and strengthening the capacity for action among the intervention area’s community, resulting in lower entomological indices. The expected distal effects were the continued control of mosquitoes, leading to a reduction in the population of both dengue and malaria vectors, as well as fewer episodes and hospitalizations related to dengue and malaria, to achieve the ultimate goal.

### Results of the fidelity analysis

Results are presented below according to the components of the intervention.

Analysis of the implementation of the components in the intervention zones (*n* = 4) revealed that operational planning was the most faithfully implemented. The other three components (community action, monitoring/evaluation, mobilization and organization) were modified (Tables 3 and 4).

**Table 4:** Implementation fidelity analysis

	Zones			
Specific descriptors	Yitouni	Tampouy-Bilbalgo	Cité Azimo	Cité An IV B
What?	Adaptations of talks and door-to-door canvassing, community activities, theatre performances Cancellation of SMS and painting Addition of drawing contest			
How?	Community approach Extensive AGIR involvement			
How often?	Adaptations in frequency			As planned
To whom?	Community residing within a 1-km radius of the Sector 20 CSPS No adaptation			
By whom?	Concurrent roles Good knowledge of the area’s resources	Concurrent roles Limited knowledge of the area’s resources	Limited knowledge of the area’s resources Three facilitators instead of four	
Contextual conditions	Influence of the political context, climatic conditions, seroprevalence study, distribution of LLINs			

#### Mobilization and organization

The mobilization and organization component was not faithfully implemented. It consisted of two elements: mobilizing and identifying community actors. All variations were related to mobilization. Members of associations and community health workers served as facilitators. They were responsible for conducting educational talks, going door-to-door, and helping to organize and mobilize the community for theatre and community activities. These facilitators were expected to work in areas they knew well and where they were accustomed to working. However, “*the challenge in identifying associations was to find associations that were really active on the ground and that were active in the four intervention zones*” [AGIR worker]. Also, the voluntary (i.e., unpaid) nature of the work annoyed certain actors. Some association members appointed by their peers to act as facilitators did not accept. This situation led to adaptations, such as assigning some facilitators to zones with which they were not familiar. The zone of the cities, the residential zones (Cité An IV B, Cité Azimo), and their potential resources were therefore not very well known to the facilitators.

*“There were people who said: I can’t go into this zone, I don’t know the zone. But because at least four people were needed per team, those people had to work in zones they didn’t know very well.”* [Yitouni facilitator].

There were also difficulties in involving some of the community leaders initially identified to participate in the implementation. For example, the people contacted at the mayor’s office and in the CSPS and its management committee (COGES) did not take part in the community intervention project in their neighbourhood.

#### Operational planning

The operational planning component was the most faithfully implemented. It consisted in validating the activities identified with community actors, developing the tools needed to carry out the activities (awareness-raising tools), training the individuals designated to carry out the activities, and drawing up a timeline. All meetings planned to select the activities were held (three in all).

*“We presented the results of the exploratory analysis... the different strategies were reviewed, and the activities that could be implemented as part of the community intervention had been really carried out, according to their perceptions, from their standpoint.”* [AGIR worker].

The training of community actors proceeded as planned. Community facilitators were trained for 3 days on dengue fever manifestations, preventive measures, and community mobilization and facilitation techniques. At the end of the training, a program for conducting activities was designed with these facilitators.

The adaptation in relation to this component had to do with the timeline. It was initially agreed by everyone that the activities should be carried out during the weekend to be able to reach more people, especially as the activities were taking place in an urban environment. Two educational talk sessions, two door-to-door outings, one theatrical performance per month, and one community activity per zone were to be undertaken. However, in the implementation, some activities were moved to working days, and the overall number of activities was increased. The facilitators conducted door-to-door outings almost daily to try to cover the intervention area. The time frame for the intervention was shortened. Instead of 6 months, the intervention took place over 4 months because the planning of activities had been done without taking into account the final entomological study to be held in October 2016 to assess the effects of the intervention.

#### Community action

Community action was not implemented as planned. This component included all activities carried out by community actors. It was the least faithfully implemented. Educational talks, door-to-door outings, community activities, and theatrical performances were held, but deviated from what had been planned.

Two activities could not be carried out. These involved putting out messages with information about dengue fever and ways to protect against the disease, and applying insecticide paint. The telephone alerts could not be carried out because “*the local operator who was supposed to transmit SMS messages to the population was unable to delineate the intervention area*” [AGIR worker 1], thereby jeopardizing the design of the impact assessment.

The drawing contest among schoolchildren in the intervention area, an activity initially discussed but not included in the operational planning, was in the end carried out.

Many adaptations were found in the implementation of activities related to this component. First, the educational talk was to be conducted with groups of people with different profiles living in the neighbourhood, so that the dengue-awareness message could be conveyed to community members. Some facilitators managed to hold a few talks with local women’s and youth associations. However, in all four zones, facilitators tried to get community members to come to a place chosen by the facilitators. As this strategy was not very successful due to low levels of community participation, the lack of intrinsic motivation of local chiefs and religious leaders, and the lack of financial incentives (especially in the urban areas), community facilitators instead met with the groups of people they encountered during the door-to-door outings.

*“With regard to the talk, we were told we could meet with people in groups, and that there was a minimum number of people to assemble. But it was hard work. So we took advantage of our door-to-door visits to talk with groups of six to seven people we met. It was the field experience that led to this modification.”* [Facilitator, Tampouy-Bilbalgo].

However, our observations revealed that the educational talks seemed to be easier to conduct than door-to-door outings. The place was identified in advance, the message was prepared, and the activity took less than an hour, whereas going door-to-door often required braving either the oppressive sun or the rain. The educational talks only required a certain amount of advance preparation, in which the community leaders were involved.

The purpose of the door-to-door activity was to check on whether the advice provided in the educational talks had been implemented. During this activity, facilitators were supposed to verify the actual destruction of breeding sites in a few randomly selected households. However, in practice, given the low level of mobilization for the educational talks, the door-to-door outreach became the primary activity of the community intervention. Facilitators systematically visited all households, transmitted messages about dengue using the images that had been intended for raising awareness in the educational talks, and identified and destroyed breeding sites.

*“We went into the houses to show household members what dengue is, to explain to them about the source of the disease, and to search for mosquito larvae and destroy them.”* [Facilitator, Cité Azimo].

Community activities in the four zones were limited to weeding days in schools and places of worship in the four areas and primarily involved schoolchildren. In the plan, the locations and the activities themselves were supposed to be chosen in collaboration with the residents of the neighbourhood. However, given the low involvement of neighbourhood residents and the difficulties in mobilizing materials, the facilitators chose to conduct the activity in schools.

*“For the community activities... the facilitators were, from the beginning, unable to take in the fact that they themselves were supposed to make contacts and to develop a social network around them that should help them work much more effectively.”* [AGIR worker 2].

With respect to the theatrical performances, the message evolved over time as the performances progressed. Initially, the message was heavily focused on the disease rather than on vector control and prevention; this was subsequently corrected with the development of a scene on methods of vector control.

#### Monitoring and evaluation

Monitoring and evaluation were not implemented faithfully. The monitoring of all activities was entrusted to an oversight committee made up of community leaders. The initial role of the committee was “*to assist the facilitators in their tasks and help find solutions to whatever difficulties might arise*.” [AGIR worker 3].

In practice, however, the monitoring of activities was modified. Of the 13 members of this committee, only one person acted in a way that demonstrated genuine commitment, and this after a meeting called by the association to try to revitalize this committee. The other members’ involvement was by and large limited to attending review meetings organized by AGIR. In the end, AGIR workers did the monitoring.

The model chosen for this dengue control intervention was the community approach [28], with community involvement throughout the implementation process. AGIR was only supposed to provide technical support to the community when the need arose. Because the implementation was not being monitored, these workers were obliged to take over the monitoring of activities, carrying out almost all the tasks assigned to the oversight committee.

*“The community stakeholders always thought AGIR [was doing everything], because from the beginning their understanding of the community approach was not quite what it should have been.”* [AGIR worker 2].

#### Specific circumstances related to context

The presence of the NGO AGIR in the Tampouy district since the last quarter of 2014 was a facilitating factor in this environment. This neighbourhood was one of five sites where passive surveillance and the household seroprevalence survey had been conducted [11]. As part of the seroprevalence survey, interviewers and CHWs visited households identified around the five CSPSs and drew blood samples from eligible persons, took entomological samples, and completed a questionnaire. With the implementation of these actions, the people living in sector 16 were somewhat familiar with AGIR and its dengue control activities, which was an asset especially for stakeholder mobilization and operational planning.

However, those earlier studies occasionally hindered the conduct of activities in this project. Some households were reluctant to receive the facilitators at home because they felt they had not been given the results of blood samples taken for the seroprevalence study.

*“In the cities, when you get there, they tell you that some people came and took blood samples and then they never heard from them again, so now they don’t want to have anything more to do with it.”* [Facilitator, Cité An IV B].

Another factor that had an impact on the implementation of the intervention was the political context. During the planning phase of the community intervention, the political situation was not conducive to the involvement of certain identified leaders. Indeed, after the communal elections, no mayor or municipal councillors were installed. This impeded any involvement of the mayor’s office in the implementation of the community intervention against dengue.

*“At first we tried to get in touch with the mayor’s office, but with the special delegation, this wasn’t at all straightforward. We were unable to involve a representative of the mayor’s office... which had an impact on carrying out community activities.”* [AGIR worker 3].

Climatic conditions (rain, sunshine) made it difficult to carry out certain activities. The location for the theatrical performances sometimes had to be moved because of sunshine, as temperatures could reach 45 degrees Celsius in the shade, and some activities were postponed because of rain. These location changes had a negative impact on attendance numbers.

*“At the first theatrical performance in our area, we had identified a place where the troupe was supposed to set up the stage, but because of the sunshine, the troupe’s manager moved the stage to a location that was near some mil beer outlets. Muslims [not at ease near a pub] refused to come.”* [Facilitator, Yitouni].

The distribution of long-lasting insecticide-treated bed nets (LLINs) for malaria control, done before the start of the community intervention, had an impact on the activities. Some people refused to receive the team of facilitators because they had not received LLINs.

The involvement of the CSPS was very subdued, which was not very helpful. Health workers most often acted as spectators. They allowed some activities to take place within the CSPS, but did not participate.

*“Collaboration with the CSPS was minimal. Ultimately, it was as if the health workers didn’t support us in implementing this community intervention.”* [AGIR worker 1].

## Discussion

To our knowledge, our study is the first to analyze the fidelity of a community-based intervention against dengue in Africa. Our results showed that the majority of the activities initially planned were carried out. However, adaptations were found in almost all components of the intervention. Results also showed discrepancies between the intervention as developed theoretically and what was actually implemented. The component that underwent the greatest adaptation was community action. For this component, activities were also added and deleted. Adaptations may have been due to the participatory nature of the intervention [18, 29], the lack of intrinsic motivation of leaders, the lack of financial incentives, and inadequate planning. These results may also have been due to an underestimation of the role of certain contextual dimensions when the intervention was being designed, such as the problem with sending SMS messages. It should also be noted that this was an intervention covering a small territory with the influence of people coming from outside the intervention area.

Community intervention is a complex process. In rural areas, community interventions generally take place in an appropriate manner [30]. Community members are willing to commit themselves to the benefit of their community, rewarded with the satisfaction of having improved their community’s well-being [30].

In urban areas, the situation is quite different. Identifying people with leadership and the ability to rally the community around an action is a real challenge [31]. In the specific case of this study, it was difficult to find community leaders and associations willing to invest themselves voluntarily and unconditionally in the fight against mosquitoes. In certain areas such as in the cities, the residential districts of Ouagadougou, the intervention designers (AGIR) had not succeeded in convincing existing associations to participate in implementing the intervention. Similar results were obtained by Espino et al. [31] in the Philippines. The implementation of their intervention suffered from not having champions to carry out the study and from a lack of ownership, which led to the failure of the intervention. Likewise in Cuba, the voluntary nature of community participation in vector control and the lack of motivation among leaders [14, 32, 33] hindered the involvement of community actors over the long term [34].

This study revealed that financial management is a real challenge for the implementation of health interventions [35] in this particular context. The lack of financial incentives led most community leaders to opt for passive observation and to conceal their monitoring role. Some facilitators looked for easy solutions that took less time and effort, with little regard for the intervention objectives. In a systematic review of community involvement in the control and elimination of communicable diseases, Atkinson et al. [36] identified financial incentives as a factor influencing participation. Yet maintaining community-based vector control requires the efforts of all stakeholders [17], which raises questions about the sustainability of these interventions in such an African urban context.

The intervention underwent adaptations that had not all been anticipated, but these did not conflict with the intervention’s change theory and respected the essential components of the intervention [37]. An adaptation is a modification of the original model of an intervention [38, 39]. The fidelity of an intervention’s implementation has an impact on its effectiveness [20, 23, 29]. Therefore, a high level of fidelity is needed to achieve the intended results [40]. In the case of this intervention, however, it certainly could not have been fully implemented or effective without adaptations. In this implementation, innovation (through adaptations) was essential for the community actors to be able to carry out the activities, as was seen in Cuba [32].

The use of participant observation has limitations, and we were aware of them. The strength of this collection method is that it is useful for obtaining data and an in-depth understanding of the subject, but there are limitations related to the impact on the analysis and interpretation of results. Triangulation of the information collected minimized this bias.

### Lessons learned

Clearly, an intervention’s context, implementation, and effectiveness are interrelated [41]. Contextual factors, and more specifically human factors (quality of work, performance, leadership, initiatives, and strategies) influence implementation [42], which explains the many adaptations observed in the community action component of this case. As such, it is important to conduct a careful preliminary analysis of the context before the intervention to identify factors that might influence the implementation (such as the actors’ motivation) and then to anticipate difficulties that might arise and work out possible solutions in a participatory way.

The community approach allows for greater ownership, but there is a need to strengthen community capacity through shared leadership, strategic planning, local communication, and behaviour change activities [43].

In our conception, participation is a collective act based on people’s volunteer efforts. We acknowledge, however, that in some contexts voluntarism might not work or would require extrinsic incentives. As argued by Atkinson, the literature shows that incentives are widely seen as inducements for participation in communicable disease control or elimination [36]. This element must be taken into account, depending on the characteristics of the settings.

The selection of community leaders is another important element to consider when designing interventions. It is important to have a broad conception of who could be a community leader. This role should not be identified only in relation to position or title, but should also take into consideration other factors, such as commitment to engage with the different groups making up the community, especially in urban areas.

## Conclusion

Dengue fever has become an inescapable reality in Burkina Faso, adding to the enormous burden of infectious diseases [11]. This community intervention was aimed at improving knowledge and filling the gap in vector control in Burkina Faso. Analysis of the fidelity of the intervention showed that most of the planned activities were carried out. However, some adaptations were necessary, and this evaluation showed especially that making adaptations can improve an intervention’s implementation and thus its effectiveness. The evaluation of the implementation fidelity of this community intervention shows its innovative character and is a crucial step in explaining the theory of intervention. From a formative standpoint, this evaluation is useful for analyzing the intervention in terms of all its components and for understanding the adaptations [44]. The results of this study can serve as a basis for future studies on the effects of the intervention and can inform decision-making regarding any scale-up.

## Abbreviations

AGIR: Action, Gouvernance, intégration, renforcement [action, governance, integration, reinforcement]
CHW: Community health worker
COGES: Comité de gestion [CSPS management committee]
CSPS: Centre de santé et de promotion sociale [health and social promotion centre]
DENV2: Dengue virus 2
DENV3: Dengue virus 3
DENV4: Dengue virus 4
IRS: Indoor residual spraying
LLIN: Long-lasting insecticide-treated bed nets
RDT: Rapid diagnostic text
SMS: Short message system
